# Everybody's Page

**Published:** 1893-01-14

**Authors:** 


### Everybody's Page.

An extraordinary claim was recently made by an American medical man. He sent in an account for 245 dollars to a lady for 47 magnetic treatments. On inquiry it appeared that the alleged treatment was given in the shape of prayers, she being resident at some distance from her medical adviser at the time. The court not seeing the possibility of countenancing such a method of treatment by a medical man, disallowed the claim. The doctor has, however, taken out an appeal.

Civilization and science which is born of it will, perhaps, some day lead to that equal diffusion amongst all classes of men of the means of cleanly and refined existence. Much has been done to purify the surroundings and make healthy the homes of our poorer citizens; but innumerable spots still remain which are neither more nor less than breeding grounds for the worst diseases which scourge the world. The lesson is always being taught, but seldom listened to, as evidenced by Hamburg.

A New York doctor gives an interesting account of a case of skin grafting on a child of one year and a half. All the fingers of the right hand were webbed together, so that the hand presented the appearance of being encased in a mitten. A clean incision separated each finger from its neighbour, and a long skin graft was applied continuously to the raw surface, down one side and up the other in each cleft. A plaster dressing covered the whole hand, and the skin grafts were left untouched for two weeks. When examined, the raw surfaces were found to be absolutely healed by the grafts, without an appearance of granulation.

It is hardly likely that the petition for the election of a Health Minister recently made to the Home Secretary by various deputations will be acceded to. It is, however, probable that the discussion of the matter and the attention brought to bear upon sanitary measures will have far more practical results than the multiplication of departments and red tapeism. Our sanitary machinery, individually, is very good. If County Councils can be brought to insist on the maintenance of uniform efficiency amongst sanitary officers it will be better still. Local officers of health have a great power of good in their hands. They should be encouraged to use it. Discretion in their election especially should be exercised, and touch with the individual officer and his district should be more strictly kept by the authorities. There is quite enough local authority in existence to maintain ordinary sanitary precautions if this is used, and this every-day sanitation is of far more value than any sudden sweeping of corners in case of emergency. The good fruits of sanitation speak for themselves. Since the sanitary laws of 1873 were passed London has decreased its mortality from 22·5 to 17·4 per 1,000 of the population. In the 17th century, when Londoners did not number one million, the mortality per thousand was 42. Therefore let hygiene be held of high importance by all means. We hold that no further unwinding of the skein will be found necessary.

There is no branch of trade in which advertisements play so important a part as in the case of patent medicines. All the doctors in the world would fail to find credence with a certain portion of the community equal to that with which quack nostrums are received. Experience has taught many of us that this is so; nevertheless, it will be a matter of surprise to learn the tale that facts in figures tell. During the past year about £240,000 was added to the revenue through the three-halfpenny stamp upon patent medicines. Considering the indiscriminate use, and abuse, we may say, of these patent medicines by the poorer classes, we are inclined to think that not only the cat amongst the inhabitants of this earth is blessed with multitudinous lives.

Mr. Fowler's scheme for the appointment of women as relieving officers of the poor has not been received with quite unanimous approval. Perhaps in this new suggestion, Mr Fowler's mind was more engrossed with the needs of those to be relieved than with the *modus operandi* themselves. The work of a relieving officer is one which would not lend itself to women of any ultra-refinement, being closely concerned with poverty in all its worst and lowest aspects. A woman who could fitly be chosen for this task, would, it seems to us, have of necessity to harden herself against her naturally compassionate and sympathetic nature, for the possession of which nature, be it observed, Mr. Fowler so pointedly commends them to the work. Though women ought to feel much indebted to Mr. Fowler for the endeavours he is making on their behalf for the extended sphere of their labour, it is but noticeable to all eyes, that in the committee he recently formed for inquiring into the administration of the Poor Law, he has not personally invited the assistance of the sex, for whose discriminating and intelligent powers he is theoretically so warm an advocate.

It is commonly accepted as a fact that the habit of drinking is certainly spreading amongst our female population of all grades. The separate entrances for women, now so general in public-houses, are looked upon partly as a trap for the more respectable and partly as an indication that the better classes of women now frequent public-houses in a way they did not heretofore. It is refreshing to find an American opinion adverse to both these theories on the subject. Dr. Crothers considers that these "ladies' entrances" are a sign of the growing ill-repute of the public-house and the desire of its patrons to conceal their visits. The same medical man attaches much importance to the restraining influence of education, amusement, and travel, on the female sex. He, however, points out the evil tendency of women drinkers to turn to drugs and narcotics, which is seldom the case in respect to men. Women are by far the greatest sufferers from the habit of drunkenness, and it is surprising that their bitter experiences do not act as effectual warnings. It must be taken for granted that the habit is one of gradual acquirement, not improbably begun in the days of courtship, when the highly objectionable custom of "treating" at the public-house first commences. Such treatings are almost as a matter of course part of any day's outings, and may certainly be regarded as the beginnings of the evil amongst the lower middle, and poorer classes at any rate.

